# Clinical Diagnosis of an Autoimmune Encephalitis Presented as a Manic Episode with Psychotic Symptoms: A Case Report

**DOI:** 10.1155/2022/2460492

**Published:** 2022-02-16

**Authors:** Miguel Soto Ontoso, Ruben A. Piernas González, Isabel Ramírez Martínez, José Miguel Meca García

**Affiliations:** ^1^Department of Psychiatry, Servicio Andaluz de Salud, Hospital de Torrecárdenas, Spain 116; ^2^Department of Psychiatry, Hospital de Poniente, Spain

## Abstract

**Introduction:**

Autoimmune encephalitis is caused by antineuronal immune mechanisms. Its clinical presentation is heterogeneous and in many cases onset with psychiatric symptoms. Paraclinical criteria guide the approach; however, the challenge occurs when there are no detectable autoantibodies in serum or cerebrospinal fluid (CSF). *Methodology*. We report one case that highlights the variability of clinical manifestations, which in the absence of antibodies was treated with immunotherapy with good response.

**Conclusion:**

In places where there is no antibody measurement, or when its measurement is negative, the clinical suspicion supported by CSF studies, magnetic resonance imaging, and electroencephalographic recording, should guide us to start immunotherapeutic treatment early. The early initiation of treatment ensures the reversibility of the neurological disorder in the vast majority of patients.

## 1. Introduction

The relationship between psychiatric illnesses and immune system dysfunctions has been studied for almost 100 years since the first autoantibodies were isolated in patients with psychosis [1]. Although since then there have been many theories and studies concerning specific immune responses to autoantigens in different psychiatric conditions, to date, we still do not have specific biomarkers due to the inconsistency of the results obtained so far [1]. If there is a syndrome with autoimmune characteristics that have attracted the attention of psychiatrists in recent years due to its clinical presentation, this has undoubtedly been autoimmune encephalitis, especially following the description of encephalitis due to N-methyl-D-aspartate receptor (NMDAR) anti-antibodies in 2005 [2]. These syndromes refer to an inflammatory disorder of the brain, of autoimmune origin, which can lead to death or the presence of significant sequelae in a high percentage of cases [3].

The exact prevalence of autoimmune encephalitis is uncertain, although recent studies estimate it at 13.7/100,000 [4]. In recent years, there has been an exponential increase in the reporting of cases, probably associated with an improvement in knowledge and methods of detection of antibodies against a specific cellular antigen [5]. It is estimated that encephalitis due to anti-NMDAR antibodies is the most frequent of all autoimmune encephalitis, even surpassing viral causes in the population under 30 years of age [6]. In a recent study, this encephalitis constitutes around 80% of seropositive autoimmune encephalitis [7].

The pathogenic hypotheses considered until not many years ago invariably spoke of a viral or paraneoplastic origin. Currently, we now know that a high percentage of these encephalitides are mediated by nonparaneoplastic idiopathic autoimmune processes. Despite this increase in the knowledge of the etiopathogenic mechanisms of these conditions, up to 50% of the cases remain without a precise etiological diagnosis [8].

These encephalitides are frequently subdivided according to whether the antigens attacked are found on the cell surface or in the intracytoplasmic space. In the former, the damage is related to cellular immunity and has a strong association with tumors (Table 1).

On the other hand, the antibodies which attack surface antigens have a pathogenic role by themselves by interfering with postsynaptic receptor signalling and leading to aberrant synaptic transmission. These encephalitides have a variable association with systemic cancer [9] (Table 2).

Although the symptomatology caused by these encephalitis varies depending on the causative antibody, the typical clinical picture includes the presence of cognitive and memory symptoms, epileptic seizures, movement disorders, and psychiatric symptoms, which reach their maximum intensity for days or weeks after onset.

The interest aroused by these encephalitides among psychiatrists lies in the fact that on many occasions their onset is with psychiatric symptoms without any other involvement of the central nervous system. It is common to find anxious symptoms, episodes of psychomotor agitation, affective disorders that may remind of manic or depressive episodes, auditory and visual hallucinations, personality changes, or delusional ideas that are usually poorly systematized. This clinical onset determines that in up to 75% of the cases, psychiatrists are the first ones to evaluate, and many times manage, these patients [10].

Until a few years ago, diagnosis hinged on the finding of antibodies in serum or cerebrospinal fluid (CSF) and the response to immunotherapy [3]. These diagnostic criteria proved to be unrealistic and could lead to a delay in the initiation of immunosuppressive treatment, with the consequent risk of allowing devastating disease progression. Many centres have limited technical access to antibody screening, antibody test results can take weeks to obtain and in almost half of the cases, and no antibodies of any kind are detected throughout the course of the disease [8]. Response to immunotherapy also appears to be a problematic aspect since there are encephalitides that do not respond to the first lines of treatment or take weeks to do so or because there are conditions such as central nervous system (CNS) lymphoma that respond to immunotherapeutic treatments [11, 12].

In an attempt to increase the accuracy and early detection of these diseases and supported by evidence suggesting that early initiation of immunotherapeutic treatment improves the prognosis, Graus et al. published a consensus a few years ago that gives the greatest diagnostic importance to the clinic and the results of conventional complementary tests accessible to most medical professionals like magnetic resonance imaging (MRI), electroencephalography (EEG), and CSF study [13, 14]. Within this standardization, there are categories with very sensitive but not very specific criteria such as “possible autoimmune encephalitis,” which can easily include conditions of primarily psychiatric origin, which limits its validity but emphasizes the importance of early initiation of immunosuppressive treatment once infectious and metabolic causes have been ruled out [13–15]. There are also more specific categories for conditions that due to their clinical presentation require their diagnostic criteria, as in the case of “definite limbic encephalitis”, “acute disseminated encephalomyelitis” or “encephalitis due to probable anti-NMDAR antibodies”. This diagnostic algorithm allows the initiation of early immunotherapeutic treatment while the results of more specific tests and antibody tests are being obtained.

First-line treatment includes tumor exeresis if present and corticosteroids, immunoglobulins, and/or plasmapheresis. Second-line treatment includes the use of rituximab and/or cyclophosphamide and/or another immunosuppressant in case of conflict with these alternatives [14]. Early use of immunotherapy treatment impacts outcome with success rates close to 80%, so its onset should not be delayed. Further studies are required to establish long-term immunomodulatory therapy [11, 16].

Here, we present a case with onset of manic and psychotic symptoms. The patient was diagnosed with limbic encephalitis of autoimmune origin based on the clinical presentation only, as no circulating antibodies were detected.

## 2. Clinical Case

### 2.1. Reason for Consultation

The patient was a 32-year-old man. He was admitted to the acute mental health unit (AMHU) because of affective exaltation and imperative auditory hallucinations, compatible with a manic episode with psychotic symptoms.

### 2.2. Personal Somatic History

The patient had dyslipidaemia, which was under hygienic dietary treatment. He had had recurrent gingival infections after a root canal two years ago. The patient started smoking at 19 years of age, but was now an ex-smoker since about 18 months. He sporadically consumed cocaine and alcohol. The patient had no known drug allergies or other somatic antecedent events or illnesses of interest.

### 2.3. Personal Psychiatric History

The patient had no previous personal psychiatric or psychopharmacological history.

### 2.4. Family History of Somatic and Psychiatric Disorders

The patient's father died of microcytic lung cancer. Four uncles in the paternal line died of neoplastic processes of various kinds (colon, pancreas, and stomach). There were no other somatic or psychiatric illnesses known in the family history.

### 2.5. Sociobiographical Background

The patient was born after an uncomplicated pregnancy and normal delivery as the youngest of two brothers. The patient had a normal psychomotor development and studied up to 8th grade. At the time when he was admitted, the patient had been married for 12 years. He lived with his wife and two daughters aged 8 years and 15 years. The patient worked as a truck driver.

### 2.6. Pharmacological Treatment

The patient was prescribed the following drugs: dexketoprofen 25 mg every 8 h and amoxicillin-clavulanic acid 875 mg every 8 h.

### 2.7. Current Illness

The patient, accompanied by his wife, attended the emergency department for the first time three days before his admission AMHU. He presented a two-week history of temporary disorientation, nervousness, verbosity, tachypsychia, emotional lability, sexual disinhibition, decreased appetite with unquantified weight loss, and insomnia. The onset of the symptomatology coincided with a dental intervention. Because of the clinical presentation, an organic screening of the patient was carried out. Basic blood tests including haemogram, ions, renal profile, liver profile and coagulation, urinalysis including detection of drugs of abuse, an electrocardiogram (ECG), and a cranial computerised axial tomography (CAT) scan were performed. Given the normality of the results of the complementary tests, the patient was diagnosed as having a hypomanic episode and admission was offered. Given the refusal of the patient and family members to admit the patient and the absence of involuntary criteria at that time, pharmacological treatment was prescribed (olanzapine 5 mg every 8 hours and lorazepam 1 mg every 8 hours) and a preferential appointment was made for outpatient mental health consultations.

Three days later, the patient returned to the emergency department accompanied by his wife. Despite taking the prescribed medication, the patient had begun to be suspicious, self-referential, and with more severe hyperthymia including episodes of intense irritability and verbal aggressiveness. In addition, he experienced complex third-person auditory hallucinations with imperative characteristics that generated intense anxiety: “They tell me to take a knife and kill.” Given the psychopathological worsening, it was decided to urgently and involuntarily admit him to the AMHU.

### 2.8. Evolution

Once on the AMHU, new blood tests were drawn, including a complete blood count, biochemistry with renal, hepatic, thyroid, ions, vitamin B12, and folic acid, and coagulation profiles. Treatment was prescribed with olanzapine 20 mg every 24 hours and lorazepam 4 mg every 24 hours, awaiting the results of the analytical results to begin the introduction of lithium carbonate. The results of the analysis were strictly normal, so treatment was started with lithium carbonate 200 mg every 24 hours.

On day 2 of admission, the patient suffered a coma episode with loss of consciousness and sphincter relaxation that required transfer to the intensive care unit (ICU), where he was orotracheally intubated, psychopharmacological drugs were removed, and levetiracetam 1000 mg/12 hours was prescribed. A cranial CAT scan was performed but no significant alterations were detected. This episode was classified as a coma, probably secondary to taking neuroleptics. Once the patient was stabilized, he was transferred again to the AMHU. From the first evaluation of the patient after his transfer, it was noted that he was disoriented in time and space and also presented a speech with gross formal alterations of thought, with a marked tendency to derailment but also including moments of incoherence. In addition, the patient presented an increasing psychomotor restlessness and intense dysphoria. Psychopharmacological drugs were reintroduced: aripiprazole 10 mg every 24 hours, valproic acid extended-release 500 mg every 24 hours and dipotassium clorazepate 45 mg every 24 hours. Levetiracetam 1000 mg every 12 hours was maintained.

During the following five days, the patient remained temporospatially disoriented and his speech was increasingly incoherent, with the appearance of thought blocks and soliloquies. Because of these symptoms, on the 7th day of admission, he was assessed by doctors from the Neurology department, who ordered an EEG, cranial MRI, and blood tests with infectious serology and autoimmune profile.

The results of the tests carried out are as follows.

EEG showed a well-organized background activity with parietooccipital alpha rhythm at 9 Hz and no evidence of paroxysmal epileptiform abnormalities. Cerebral MRI did not show any significant alterations. Regarding the results of the blood test, see Table 3

While awaiting the autoimmunity profile, the neurologist recommended maintaining the same treatment and observing the clinical evolution of the patient.

During these first seven days of admission, the patient had had two hypertensive crises (blood pressure (BP) 180/110 mmHg and 170/110 mmHg) that had been treated symptomatically.

The following day, the 8th day of admission, fever (37.4°C), elevated blood pressure (155/110 mm Hg), O_2_ saturation 95%, and sinus tachycardia at 115 bpm was observed. The patient began to show fluctuations in the level of consciousness reaching stupor, without maintaining eye contact, unable to obey simple commands, with decreased response to painful stimuli, presenting chewing movements and incoherent language. Urgent consultation with the internal medicine department was made, and a new blood and urine analysis was performed, as well as urine and blood cultures. The results of the complementary tests showed increased leukocytosis with neutrophilia (15.28 and 11.98 × 10^3^/*μ*L, respectively), an international normalized ratio (INR) of 1.19 and a D-dimer of 4902 ng/mL. Acute phase reactants were similar to the previous days. The results of the urine and blood cultures, obtained days later, were negative. Computerized tomography (CT) angiography confirmed the presence of a thrombus in the distal basal segmental artery of the left lower lobe. The patient was transferred to an internal medicine ward due to decreased level of consciousness and febrile syndrome. Empirical antibiotic therapy was started with amoxicillin-clavulanic acid 1000 mg every 8 hours, and psychotropic drugs were withdrawn.

Although there was no clear semiology of meningeal irritation in the physical examination, the presence of fever without focus and the deterioration of the level of consciousness made it necessary to rule out inflammatory meningoencephalic involvement so a lumbar puncture (LP) was performed. The LP showed pathological results: red blood cells 2000/mm^3^, leukocytes 47/mm^3^, polymorphonuclear 10%, monocytes 80%, proteins 0.27 g/dL, and glucose 93 mg/dL with a blood glucose of 115 mg/dL. The patient was diagnosed with limbic encephalitis of probable infectious or paraneoplastic origin given the patient's recent oral surgery and the family history, respectively. Empirical treatment with acyclovir 10 mg/kg every 8 hours was prescribed, and the patient was transferred to the neurology department.

A battery of complementary tests was requested for etiological affiliation of the symptomatology: polymerase chain reaction (PCR) virus and neurotropic bacteria (Escherichia coli, Haemophilus influenzae, Listeria monocytogenes, Neisseria meningitidis, Streptococcus pneumoniae, Cytomegalovirus, Enterovirus, Herpes simplex virus type 1 and 2, Human herpesvirus 6, Parechovirus, Varicella-zoster virus, and Cryptococcus neoformans), and protein 14.3.3 in CSF, echocardiography, abdominopelvic CAT, and positron emission tomography (PET). All the results were normal so paraneoplastic, and infectious aetiology was ruled out.

Given the high possibility of autoimmune aetiology, on day 10 of admission, acyclovir was withdrawn and treatment was started with methylprednisolone 1 mg/kg every 24 hours and inmunoglobulin 0.4 mg/kg/every 24 hours.

On the 12th day of admission, the patient suffered three episodes of coma with disconnection from the environment, tongue biting, and decreased O_2_ saturation. The patient was again transferred to the ICU where he required orotracheal intubation, mechanical ventilation, and sedation with propofol, midazolam and fentanyl. Anticomituitary treatment with lacosamide, phenytoin, and valproic acid was indicated. After seven days of sedation, day 19 of admission, due to the poor evolution of the patient, seven sessions of plasmapheresis were started with a good clinical response. The clinical improvement of the patient allowed the progressive withdrawal of sedation and mechanical ventilation, and he was discharged from the ICU after 35 days of admission (day 47 of admission). He was transferred again to the Neurology department, where he continued to be admitted for 45 more days, during which the patient's evolution towards improvement was slow but progressive. During these days, the anti-inflammatory treatment was adjusted downwards. The psychiatry service carried out a joint follow-up of the patient, gradually appreciating a better orientation in space and time and a language becoming progressively more organized, with the disappearance of the blocks and other formal disorders. At the affective level, he was euthymic. However, he began to verbalize a deliroid ideation focused on his wife, which moderately pressured the patient. It was decided to prescribe ziprasidone until reaching a dose of 60 mg every 24 hours, resulting in a degradation of the pressure exerted by these ideas.

After 92 days of hospitalization, the patient was finally discharged home. At the time of discharge, the patient was oriented in space and person and partially oriented in time. Short-term memory was slightly affected. There was no psychomotor impairment. Speech was formally well-constructed. However, he presented a poorly systematized delusional idea centered on his wife; these ideas were reduced under pharmacological treatment but did not completely disappear. There were no alterations in the sensory-perceptive sphere. The patient was euthymic, and no anxiety was observed. He slept well, and there was no appetite disturbances, no ideas of death, and no suicidal ideation. Reality judgement was preserved.

### 2.9. Pharmacological Treatment at Discharge

When discharged from the hospital, the patient was prescribed the following drugs: ziprasidone 20 mg every 8 hours, omeprazole 20 mg every 24 hours, brivaracetam 150 mg every 12 hours, lacosamide 200 mg every 12 hours, prednisone 90 mg every 24 hours, enoxaparin 80 mg every 24 hours, and azathioprine 50 mg every 12 hours.

## 3. Discussion

Although the relationship between immune function and behaviour and cognition has been hypothesized for many decades, it has not been until recent years that the most evidence and knowledge in this direction has accumulated [17–19]. The improved clinical and diagnostic characterization of autoimmune encephalitis and the increased ability to detect the causative antibodies have played a fundamental role in this. These encephalitides have made it possible to demonstrate the relationship between the attack on proteins involved in synaptic transmission and the triggering of severe psychiatric symptoms. Perhaps the paradigmatic example is the autoimmune encephalitis caused by antibodies against the NMDA receptor, both for being the most frequent and for presenting psychiatric symptoms in 95% of cases [20, 21].

The clinical recognition of these encephalitides is fundamental for psychiatrists. As it happened in the clinical case presented, it is very frequent that the first professionals who evaluate this type of symptoms belong to mental health organisations. This can be explained by the fact that their onset may be with psychiatric symptoms only and because some of them, including the most frequent of all, encephalitis due to anti-NMDAR antibodies typically occur in young people between the 2nd and 4th decade of life. This age range corresponds precisely with the highest peak incidence of psychosis onset. Another reason why psychiatrists should include this syndrome in the differential diagnosis of primary psychosis is that these are conditions that, if left untreated, can lead to serious sequelae or death. It is this potential risk of serious health compromise that determines the need for early recognition of these syndromes. However, the early diagnosis of this type of symptoms is not simple as we can see in the clinical case presented, in which 10 days passed from the appearance of the first symptoms until immunosuppressive treatment was started.

Until the publication of the diagnostic consensus of Graus et al., the diagnosis of this syndrome needed to determine the causative antibodies. This is risky and not very operative because it could lead to a delay in the start of the treatment since many health centres may have to send biological samples to reference laboratories that may take several weeks to send the results, and because in up to 50% of autoimmune encephalitis, it is not possible to isolate the causative antibody [8]. Trying to obviate the need for specific antibodies for the diagnosis of this syndrome, Graus et al. give a fundamental role to the clinical presentation and the findings in the complementary tests, leaving the determination of antibodies as a test to support the clinical diagnosis [11]. The use of antibody panels with broad antibody profiles relevant to encephalitis in serum and CSF is preferable to clinician-selected individual tests because the clinical phenotypes of these syndromes overlap, new antibodies are discovered every year, and also there is a possibility that more than one antibody is present.

The clinical case presented serves as an example that if the classic diagnostic criteria had been followed, the patient could not have been diagnosed and treated adequately since the antibodies causing the condition were never identified.

The typical clinical presentation of these conditions consists of rapid development of a confusional syndrome, a subacute development of a working memory deficit, psychiatric symptoms with frequently atypical presentations, and often seizures. The subacute development of short-term memory loss is considered the hallmark of the disorder but may be overlooked due to the presence of other symptoms [21, 22].

Psychiatric symptomatology is often the first to appear because at the onset of the disease the concentration of antibodies is low and serious tissue damage has not yet occurred. Depressive or manic mood, unspecific fears, anxiety, insomnia, sensory-perceptual disturbances, or delirious ideas, usually scarcely systematized, may appear [21, 23, 24].

The presence of generally atypical psychiatric symptoms, with poor response to neuroleptics [25], in addition to seizures, confusion, and short-term memory loss, should lead us to include this syndrome in the differential diagnosis.

The complementary tests recommended for its diagnosis are the study of CSF and the performance of an EEG and a cranial MRI with contrast, in addition to those required to locate the tumor and rule out infectious-metabolic aetiology [12].

The EEG may not present alterations or these may be nonspecific, although we can also find a focal or diffuse slow or disorganized activity or epileptic activity. In some encephalitis, there are characteristic patterns, such as the delta brush rhythm in encephalitis caused by anti-NMDAR antibodies [24, 26].

MRI often shows increased signal on T2 FLAIR images on the medial aspect of the temporal lobes, although there may also be no findings or unilateral temporal involvement [27].

The CSF study in autoimmune encephalitis can show abnormal results in up to 80% of cases [11]. The most frequent findings are lymphocytic pleocytosis (90%), hyperproteinorrachia (33%), and the presence of oligoclonal bands (25%) [28, 29]. However, these are nonspecific findings [15]. A CSF without inflammatory changes would indicate with a high degree of certainty the existence of a noninflammatory encephalopathic condition, although the autoimmune aetiology cannot be completely ruled out and should continue to be studied as a probable aetiology [30, 31].

In our patient, the presentation of autoimmune encephalitis was with psychiatric symptoms compatible with a manic episode with psychotic symptoms. As in any first psychotic episode, an organic screening was carried out in the emergency department, and no alterations were detected in the routine complementary tests performed (cranial CT scan, blood count, biochemistry, and urinary sediment) that would point to an organic origin of the symptoms. Once the patient was admitted, given the atypical nature of the psychiatric symptoms and the appearance of symptoms typical of a CNS disorder (decreased level of consciousness, seizures), it was decided to perform an LP whose result was indicative of inflammatory encephalopathic pathology. In a decision that probably saved the life of the patient and protected him from new neurological sequelae, once the infectious, metabolic, and paraneoplastic causes had been reasonably ruled out, it was decided to start immunosuppressive treatment. This decision was made based on the symptoms and the nonspecific results of the LP, although no antibodies were detected in the patient and there were no alterations in any of the other complementary tests performed (EEG, brain MRI).

The clinical case we have presented illustrates the importance for psychiatrists of knowing the clinical characteristics and forms of presentation of autoimmune encephalitis. The correct and early diagnosis, mainly clinical but supported by standard complementary tests, will allow the early establishment of the appropriate treatment for these conditions, an aspect that can make the difference between life and death for the patient.

## Figures and Tables

**Table 1:** Main antibodies against intracellular antigens associated with autoimmune encephalitis.

Antibody	Associated syndrome	Tumor frequency	Tumor type
Anti-Hu	Limbic encephalitis, encephalomyelitis, sensory neuropathy, cerebellar degeneration, autonomic neuropathy [10]	>90%	Small-cell lung cancer, neuroblastoma, prostate cancer
Anti-RI	Romboencephalitis, myoclonus opsoclonus, cerebellar degeneration [10]	>90%	Small-cell lung cancer, gynaecological tumors, breast cancer
Anti-Ma	Limbic encephalitis, rhomboencephalitis, cerebellar degeneration [32]	>90%	Testicular tumors, small-cell lung cancer, breast cancer
Antiamphiphysin	Stiff-man syndrome, Lamber Eaton syndrome, motor-sensory polyneuropath, encephalomyelitis, paraneoplastic cerebellar degeneration. [33]	>90%	Breast, small-cell lung cancer
Anti-CV2/CRMP5	Limbic encephalitis, encephalomyelitis, optic neuritis, cerebellar degeneration, sensory-motor neuropathy, chorea [34]	>90%	Small-cell lung cancer, thymoma
Anti-GAD65	Limbic encephalitis: prodominant temporal lobe epilepsy with moderate cognitive impairment. It is also associated with stiff person [35].	Not associated	Associated with other autoimmune diseases such as diabetes and thyroiditis
GFAP-IgG	Autoimmune meningoencephalomyelitis, Autoimmune astrocytopathy [36]	20%	Teratoma, adenocarcinoma

CRMP5: collapsin response mediator protein; 5 GAD: glutamic acid decarboxylase; GFAP-IgG: glial fibrillary acidic protein immunoglobulin G.

**Table 2:** Main antibodies to surface antigens associated with autoimmune encephalitis.

Antibody	Associated syndrome	Tumor frequency	Tumor type
NMDA receptor	Anti-NMDA receptor encephalitis [37]	40%	Teratoma
AMPA receptor	Limbic encephalitis [38]	65%	Thymoma, small-cell lung cancer
GABAa receptor	Encephalitis with refractory seizures, status epilepticus [39]	25%	Thymoma
GABAb receptor	Limbic encephalitis with predominance of epileptic seizures at onset [40]	50%	Small-cell lung cancer
LGI1	Limbic encephalitis, frequent hyponatremia [41]	10%	Thymoma
CASPR2	Morvan syndrome; limbic encephalitis [42]	Overall, 20%; in syndrome of Morvan, 20-50%.	Thymoma
DPPX	Encephalitis, hyperecplexia [43]	<10%	Lymphoma
mGLUR5	Encephalitis [44]	55%	Hodgkin's lymphoma
Neurexin 3alpha	Encephalitis [45]	0%	—
Dopamine D2 receptor	Encephalitis of basal ganglia (dystonia, parkinsonism, chorea, oculogyric crises) [46]	0%	—
Glycine receptor	Progressive encephalitis with rigidity and myoclonus, s t i ff- person syndrome, encephalitis [47]	<5%	Thymoma, lung, Hodgkin's lymphoma
Aquaporin 4	Encephalitis [48]	0%	—
MOG	Acute disseminated encephalomyelitis [49]	0%	—
GQ1b	Bickerstaff's brainstem encephalitis [50]	0%	—
IgLON5	Nonrapid eye movement (REM) and REM sleep disorder, brainstem dysfunction [51]	0%	—

NMDA: N-methyl-D-aspartate; AMPA: *α*-amino-3-hydroxy-5-methyl-4-isoxazole propionic acid; GABAa: *γ*-aminobutyric acid type A; GABAb: *γ*-aminobutyric acid type B; LGI1: leucine-rich glioma inactivated 1; CASPR2: contactin-associated protein 2; DPPX: dipeptidyl-peptidase-like protein-6; mGluR: metabotropic glutamate receptor; MOG: myelin oligodendrocyte glycoprotein; GQ1b: tetrasial ganglioside; IgLON5: immunoglobulin-like cell adhesion molecule 5.

**Table 3:** Results of blood tests, serology, and autoimmunity.

TEST	Abnormal results	Normal results
Blood test	CBC: leukocytes 11.81 × 10^3^/*μ*L, neutrophils (count) 9.40 × 10^3^/*μ*L, neutrophils (percentage) 79.62%, lymphocytes (percentage) 12.01% Biochemistry: gamma-glutamyltransferase 110 U/L, alanine transaminase 81 U/L C-reactive protein 8.5 mg/L	Vitamins including B12, folic acid and vitamin D, and the rest of results of CBC and biochemistry Normal thyroid profile Normal clotting
Serology (obtained 5 days after analytical extraction)	—	Negative for active infection of syphilis, cytomegalovirus, human immunodeficiency virus, hepatitis B and C virus, herpes simplex virus 1 and 2, and varicella-zoster virus.
Autoimmunity	Weak positive for antinuclear antibodies (1/80) with a speckled pattern.	Immunoglobulin A, G, M, complement C3 and C4 Negative for antibodies: (i) Anti-double-stranded DNA (ii) Extractable nuclear antigen (including anti-RNP UI (70 kDa+A+C), anti Sm, anti-SSA/Ro, anti-SSB/La, anti-Scl-70, anti-Jo-1) (iii) Anticardiolipin (iv) Anti-beta-2-glycoprotein I (v) Antineutrophil cytoplasm (vi) Antitransglutaminase (vii) Antithyroid peroxidase (viii) Anticyclic citrullinated peptide (ix) Rheumatoid factor (x) Antineuronal (intracellular antigen): antiamphiphysin, antineuronal-CV2, anti Ma1, anti Ma2, anti-Hu, Ac anti-Ri, anti-Yo, anti-GAD (xi) Anti-NMDAR

## Data Availability

The clinical case data used to support the findings of this study are restricted by the GDPR in order to protect patient privacy. Data are available from Miguel Soto Ontoso (miguelsotoontoso@hotmail.es) for researchers who meet the criteria for access to confidential data.
